# 

**Published:** 1988-02

**Authors:** 


**Editor**
Mr M. G. Wilson
*40 Coombe Lane, Bristol BS9 2BJ*
*Tel: 0272-682320*

**Assistant Editor**
Dr P. R. Goddard

**Editorial Committee**
Mr B. P. Jones (Secretary),
*Medical Librarian, Bristol University*
Dr J. L. Burton

**Correspondents**
Dr A. E. Adam (Taunton)
Mr D. C. C. Bartolo
Mr M. L. Crosfill (Penzance)
Dr J. D. Davies
Dr J. Jancar
Professor D. J. Pereira Gray (Exeter)
Dr H. G. Mather
Professor G. M. Stirratt
Mr C. Teasdale (Plymouth)
Dr J. L. G. Thomson
Dr M. J. Whitfield
Professor G. K. Wilcock

**BRISTOL MEDICO-CHIRURGICAL SOCIETY**

**President**
Dr H. M. Norman

**Secretary**
Mr R. N. Baird,
*23 Old Sneed Park, Bristol BS9 1RG*

**Treasurer**
Dr N. C. Tricks,
*The Mill, Wrington, Bristol*

**Published by**
**Clinical Press,**
*Greinton House,*
*8 Exeter Buildings,*
*Redland,*
*Bristol BS6 6TH*

**Typesetting by**
*Avon Communications Ltd.,*
*Avon House, Nailsea, Bristol BS19 2DJ*

**Printed by**
*Suttons, Paignton*

Established 1883

### BRISTOL MEDICO-CHIRURGICAL JOURNAL

**FEBRUARY 1988**
**Volume 103 (i)**

Copyright © Clinical Press Ltd 1988.

Published quarterly and distributed free to all doctors in the West of England. Overseas subscription £12.

**NOTICE TO CONTRIBUTORS**
Contributions are invited especially from West of England authors on a variety of subjects, e.g. Original articles relating to the practice of medicine, reports on the contemporary medical scene, obituaries, historical accounts, recollections of colleagues and events worthy to be remembered and liable to be forgotten. An article of 2,000 words with 2 illustrations will occupy 2 pages and this is a suggested, though by no means an obligatory length. Articles are preferred in the form proposed by the International Committee of Medical Editors (Vancouver System)—pamphlet obtainable from Editor of BMJ., BMA. House, Tavistock Square, London, WC1H 9JR.

### The Bristol Medico-Chirurgical Society

The Society invites doctors in the Bristol area to join, and reminds existing members of the need for regular recruitment and asks them to invite colleagues who they think would enjoy membership.

The society was founded in 1874 with fifty members. Regular meetings have been held ever since without interruption by two world wars. Initially, as now, papers were given, there were discussions on chosen themes and case presentations. From its inception the society has been a 'club' at which doctors from Bristol and the surrounding area could meet together, it formed a bond between doctors in general practice, hospital doctors, doctors in the public services and others. This is still the most important function of the society. In the early days it was perhaps the main source of postgraduate education, nowadays there are meetings, conferences and courses on all subjects aplenty and the Postgraduate Centres are a constant source of further education. Accordingly meetings of the society are designed less to educate than to interest, and speakers well known in other fields are invited. The social aspect is enhanced by a buffet supper. Most of the meetings are held in the postgradute centres, but there are also visits with clinical meetings at local hospitals or to local places of interest. New members are usually recruited by existing members, but any doctor wishing to join who does not know who to approach should write to the Hon. Secretary, Mr Roger Baird, 23 Old Sneed Park, Bristol BS9 1RG who will be glad to find a proposer. The society is particularly glad to welcome young members.

